# Exploring public health education’s integration of critical race theories: A scoping review

**DOI:** 10.3389/fpubh.2023.1148959

**Published:** 2023-04-14

**Authors:** Sarah L. Collins, Travis C. Smith, George Hack, Michael D. Moorhouse

**Affiliations:** ^1^College of Public Health and Health Professions, University of Florida, Gainesville, FL, United States; ^2^Higher Education Administration, Department of Educational Foundations, Leadership, and Technology, College of Education, Auburn University, Auburn, AL, United States; ^3^Department of Occupational Therapy, College of Public Health and Health Professions, University of Florida, Gainesville, FL, United States

**Keywords:** public health, higher education, curriculum, instructional strategies, critical race studies, scoping review

## Abstract

**Introduction:**

Public health has declared a commitment toward diversity as a whole, with a commitment toward addressing and dismantling racism being at the forefront. Although public health has admirably taken on this mission, and the foundational principles of public health align with social justice and health equity, public health as a discipline is vastly behind other fields in integrating and utilizing critical race theorizations. Of particular concern is the lack of critical race theorization within public health education materials. Public health education serves as a precursor to public health practice and situates topics and competencies that are essential to one’s foundational public health knowledge and skillset, thus the use of strong theoretical groundings is critical in public health education.

**Objectives:**

Therefore, to explore the current landscape of public health educational research that employs critical race theories, this study sought to conduct a scoping review investigating the current literature of public health pedagogical, instructional, and curricular efforts that utilize race and antiracist theorization principles as a means to administer public health education. More specifically, we sought to investigate how have faculty and instructors published their integration of race theorization in public health curriculum/instruction within the United States since 2011.

**Results:**

We found 18 examples from peer-reviewed literature of curricular, pedagogical, or instructional practices and strategies that integrate critical theories of race, including contemplative pedagogy (*n* = 1), antiracism (*n* = 3), Public Health Critical Race praxis (*n* = 4), Critical Race (*n* = 5), critical service-learning/community engagement (*n* = 2), ethnic studies (*n* = 1), and intersectionality (*n* = 2).

**Conclusion:**

These articles present a wide breadth of innovative approaches to infusing critical race studies within public health higher education, ranging from individual assignments to course design and implementation to institutional culture change, thus demonstrating the multifaceted nature of critical race studies within micro-learning communities and macro-discipline practices. Identifying theoretically grounded, exemplary models and scholarly recommendations of pedagogical, instructional, and curricular practices provides readers the opportunity to borrow from successful practices and implement concepts of race, racism, antiracism, intersectionality, and more into their classrooms.

## 1. Introduction

In recent years, racism has been declared a public health crisis (1). Public health entities such as Healthy People (2), American Public Health Association (3), and Center for Disease Control and Prevention (4) have declared a commitment toward eliminating health disparities, achieving health equity, and attaining social justice for all populations. These coinciding missions ultimately hope to attend to public health’s nationally identified essential services, which center equity in an effort to “enable good health and seek to remove obstacles and systemic and structural barriers, such as poverty, racism, gender discrimination, and other forms of oppression” (5).

Though there has been a commitment toward diversity as a whole, a commitment toward addressing and dismantling racism has been particularly at the forefront. Racism can be defined as “organized systems within societies that cause avoidable and unfair inequalities in power, resources, capacities and opportunities across racial or ethnic groups. Racism can manifest through beliefs, stereotypes, prejudices or discrimination” (6). Within public health research, the harmful relationship between racism and health that burdens ethnic and racial minority populations is well documented (6–11). Evidence suggests that this relationship exceeds interpersonal accounts of racism, since it is also reflected within institutional, systematic, and cultural aspects of American society (12). The expansive nature of racism and discrimination within society has ultimately catalyzed these national public health agencies to prioritize abolishing and dismantling systems of oppression related to race in recent years.

Although public health has admirably taken on this mission, and the foundational principles of public health align with social justice and health equity, public health as a discipline is vastly behind other fields. Fields such as education and law have dealt in theorizing racial frameworks and concepts within discipline-specific socioenvironmental and sociocultural contexts for decades, whereas public health is only recently engaging in these conversations. Much of public health’s critical frameworks, such as the Social Determinants of Health, discuss race and ethnicity as a variable to depict health disparities and health inequities across populations rather than acknowledging that *racism* permeates through all institutional, structural, and societal entities within the United States. By focusing on racial and ethnic indicators, we continue to perpetuate race and ethnicity as proxy measures for racism, rather than reifying racism itself. Similarly, efforts such as Alang and colleagues (13) call for public health practitioners to dismantle White supremacy are well intentioned but lack a theoretical grounding. Theoretical frameworks are meant to serve as guides of research and practice, which ultimately informs all efforts starting from topic selection to design approach to evaluation plan (14). The integration and use of theories and theoretical frameworks is significant in establishing clear direction, systematic procedures, and methodologically sound practices for others to contextualize one’s work and translate it into their respective field. Currently, the presence of literature that infuses critical race theories or antiracist praxes within public health is limited.

Broadly, traditional theories seek to present interrelated constructs or concepts relevant to a specific phenomenon in an effort to either explain or predict variable outcomes (15). In contrast, critical theories seek to explore central features and characteristics of contemporary society and critique it as a whole to catalyze social transformation (16, 17). Within critical studies of race, researchers, scholars, and practitioners object to the experience of White individuals and Whiteness being a normative standard, and instead seek to center the experiences of people of color and racial oppression through alternative methodologies such as literary narrative knowledge and storytelling (18). It should be emphasized here that not all theories that consider race are consider *critical*. Criticality refers to naming and analyzing structural forms of oppression, objecting to their ordinariness, and challenging the status quo through action and social transformation (19, 20). Among the most widely known critical race theorizations is Critical Race Theory (CRT).

The 1960s civil rights era catalyzed the development of Critical Legal Studies (CLS), as lawyers, activists, and legal scholars collectively proposed theories and strategies to combat both the overt and subtle forms of racism that permeated the United States (21). However, grounded in philosophical differences, critical race studies emerged in the 1970s as critical race scholars sought to combat racism through the existing legal channels rather than abandon the notions of legal rights altogether; thus a new thread of critical race studies was born (22), and CRT was formally recognized in 1989. As such, CRT not only represents an assortment of theoretical constructs, but also embodies a collective movement among activists and scholars committed to exploring and transforming the relationship among race, racism, and power to transcend current racial relationships and reach a utopia of an antiracist society (21). Though CRT is the most widely recognized critical race theory, several others have emerged from various disciplines and scholarship.

As previously mentioned, though critical race theories are actively present in conversations across interdisciplinary engagements, public health has been slow to engage in this scholarly discourse. In 2010, Ford and Airhihenbuwa (23, 24) acknowledged that racism within public health frameworks is often disconnected from racial theorization, and therefore proposed the first public health framework that infuses Critical Race Theory: The Public Health Critical Race (PHCR) praxis. As a result, several researchers have utilized this praxis as a theoretical underpinning of their work (25–28). Coincidentally, the release of the germinal PHCR praxis coincided with an amendment to the Council of Education for Public Health (CEPH) accreditation criteria which called for researchers and educators alike to situate race and racism-related consequences at the forefront of their efforts (29, 30).

This was further fleshed out upon the release of the 2016 criteria standards, which included a specific competency to “discuss the means by which structural bias, social inequities and racism undermine health and create challenges to achieving health equity at organizational, community and societal levels” (31). However, unlike the response to the PHCR praxis, there is little to no research demonstrating implementation of critical race studies in public health education to meet these criteria. Public health education serves as a precursor to public health practice and situates topics and competencies that are essential to one’s foundational public health knowledge and skillset; therefore, the lack of theoretically grounded literature within public health education is alarming. Therefore, to explore the current landscape of public health educational research that employs critical race theories, this study seeks to conduct a scoping review investigating the current literature of public health pedagogical, instructional, and curricular efforts that utilize race and antiracist theorization principles as a means to administer public health education.

## 2. Methods

Scoping reviews are a popularized approach to synthesizing published literature and research evidence to examine the variety, breadth, and nature of a specific topic area or research question (32–34). This methodological approach is specifically advantageous when there is a gap in the literature due to limited investigation, high complexity, or heterogeneity in findings (34, 35). Arksey and Malley (32) presented one of the original scoping review methodological guidelines and have since served as the gold standard in scoping review procedures. As such, this scoping review will follow the authors’ proposed framework, which provides five procedural stages: (1) identifying the research question, (2) identifying relevant studies, (3) study selection, (4) charting the data, and (5) collating, summarizing, and reporting the results (32).

### 2.1. Identifying the research question

Unlike systematic reviews, scoping reviews seek to obtain a comprehensive overview of available literature regarding a specific research area regardless of study design (32). One specific aim of scoping reviews is to explore the breadth and nature of published research (32), which may inherently identify a gap in the existing literature. As it currently stands, there is limited published literature within public health that explicates educational utilization of critical race theorization. Furthermore, in response to CEPH’s call for accredited schools and programs of public health to demonstrate a commitment to diversity (29, 30), as well as Ford and Airhihenbuwa’s (23) global call for public health researchers, practitioners and scholars to engage in the PHCR praxis, this scoping review asks: Since 2011, how have faculty and instructors published their integration of race theorization in public health curriculum/instruction within the United States?

### 2.2. Identification of relevant studies

A comprehensive search was conducted within the following databases: PubMed, Web of Science, ProQuest, and EBSCO. Within ProQuest the following databases were searched: Black Studies Center, Ethnic NewsWatch, Social Science Premium Collection’s Education Collection, Social Science Database, and Sociology Collection. Within EBSCO, the following databases were searched: Academic Search Premiere, APA PsycInfo, Chicano Database, CINAHL, Education Source, Health Source: Nursing/Academic Edition, Humanities Source, Professional Development Collection, Psychology and Behavioral Sciences Collection, Race Relations Abstracts, and Sociology Collection.

The following sequence of search terms was reviewed within the title and abstract fields within each database: “public health” AND (“critical race theory” OR CRT OR “race theory” OR race OR racism OR racialization OR racialism OR decolon* OR antiracism OR “antiracism”) AND (curricul* OR instruct* OR train* OR teach* OR taught OR educat* OR faculty). Search procedures were conducted in January 2022. After screening the above databased, a total of 2,787 articles were identified and imported into EndNote 20 (36). The resulting EndNote library was then compressed into a compatible file to upload to University of Florida’s (UF) Covidence software system (37), due to its reported high usability score and preferability among researchers (38). Upon import, 1,546 duplicates removed, leaving 1,242 articles for title and abstract screening.

### 2.3. Study selection

Literature suggests that eligibility criteria should be iteratively reviewed once researchers have an opportunity to familiarize themselves with the nature of the literature (33). Therefore, the eligibility criteria were amendable, if needed. The finalized inclusion criteria included articles that: (1) were published in English, (2) were from academic, peer-reviewed journals (3) had full text available, (4) focused on theorization of race in public health curriculum, instruction, or teaching, and (5) solely included study sites within institutions of higher learning (i.e., universities, colleges, and community colleges). Studies were excluded if (1) they were legal articles, dissertations, gray papers, non-peer-reviewed journal articles, books, or reports, (2) the study site was not exclusively based in the United States, (3) the study did not operationalize race within public health curriculum, instruction, or teaching, (4) the study explored race strictly as a demographic or organizing variable, (5) the study occurred in a pre-K-12 setting, (6) the study only provided calls-to-action for public health education rather than providing insight into their specific grounding or integration of race theorization, or (7) the study did not provide instructor and/or faculty perspective (i.e., exclusively gave a student perspective). Furthermore, only studies published after June 2011 (when CEPH’s amended report was published) until present were included.

The above eligibility criteria were applied during two sequential rounds of screening. First was the title and abstract screening, which has been found to swiftly, yet methodically, reduce large quantities of articles (39). At this stage, 1,108 articles were excluded, with detailed notes for each article stating their reason for exclusion. The second round consisted of a full-text review, which allows researchers to get a more comprehensive understanding of the published work compared to the previous stage. During this round, 116 articles were excluded, with reasons for exclusion listed for each article. A second reviewer screened the excluded articles at each stage, noting any discrepancies in their review. No discrepancies were identified.

**Figure 1:**
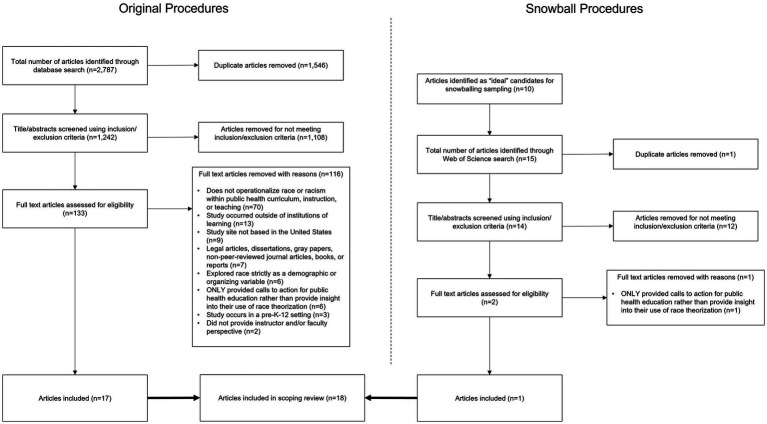
Article selection process.

Upon the conclusion of full-text reviews, the lead researcher identified any articles that depict an “ideal” candidate to conduct snowball sampling. An “ideal” candidate was an article that near perfectly aligned with the research aim of this scoping review. Ten articles were identified as “ideal.” Articles that cite these 10 “ideal” candidate articles, identified through Web of Science’s advanced search features, were screened using the same eligibility criteria outlined above. Similar to the original screening, two researchers reviewed any articles excluded from the abstract/title screening and full-text review. Fifteen articles were identified from the snowball sampling approach and imported into EndNote. Once compressed and added to UF’s Covidence, one duplicate was removed, leaving 14 articles for title and abstract screening. Twelve articles were excluded during the title and abstract screening and one article was excluded during the full-text review. A visual representation of the article selection process can be found in Figure 1.

### 2.4. Charting the data and summarizing results

This scoping review sought to extrapolate the following data from the resulting articles: title of manuscript, manuscript type, author, year of publication, region/state, reported critical race theorization, aims/purpose, key characteristics findings, and author (s)’ race (if specified) and positionality (if provided). Researchers argued that research is not strictly objective, but rather personal in that one’s position is ever evolving within shifting social networks, relationships, and dynamics (40–42). As such, it is understood that the researcher plays a central role in the research process (42), thereby influencing research dynamics, analysis procedures, and disseminated results. Therefore, only explicit statements regarding one’s position within social, political, ontological, or epistemological contexts were included for author(s)’ positionality. The lead researcher conducted the data extraction and collated the results according to thematic similarities within reported critical race theorization.

## 3. Results

Of the 116 articles excluded from the original full texts reviewed, a majority did not operationalize race or racism, or present within the context of, public health curriculum, instruction, or teaching (*n* = 70). Additional reasons for exclusion included the study occurred outside of institutions of higher learning (*n* = 13), the study site was not based within the United States (*n* = 9), the texts were legal articles, dissertations, gray papers, non-peer-reviewed journal articles, books, or reports (*n* = 7), the article explored race strictly as a demographic or organizing variable (*n* = 6), it only provided a call to action rather than insight into how to integrate or employ race theorization (*n* = 6), the study occurred in a pre-K-12 setting (*n* = 3), and it did not provide an instructor and/or faculty perspective (*n* = 2). Of the snowball sampled articles, the one article excluded from the full text reviewed was due to only providing a call to action rather than insight into how to integrate or employ race theorization.

Accounting for both original screening efforts and snowball sampled efforts, 18 articles were included in this scoping review. Among the 18 articles, a variety of critical race theories and frameworks were employed as a means to provide a theoretical grounding in the reported public health instructional, curricular, and pedagogical efforts. These include contemplative pedagogy (n = 1), antiracism (*n* = 3), Public Health Critical Race (PHCR) praxis (*n* = 4), Critical Race Theory (i.e., racism, centering the margins, power differentials, etc.) (CRT, *n* = 5), critical service-learning/community engagement (*n* = 2), ethnic studies (*n* = 1), and intersectionality (*n* = 2). Articles were organized according to their most prominent theorization and/or framework, however, it should be recognized that a significant portion of the included articles leaned on more than one theory and/or framework in their reported efforts.

Interestingly, for identified articles with a specified location, they congregated within only a few geographical areas including North Carolina (*n* = 3), Maryland (*n* = 2), California (*n* = 2), Washington (*n* = 2), Michigan (*n* = 1), and Rhode Island (*n* = 1). The remaining articles either did not provide a specific location or stated a general region such as the Northeast (*n* = 1) or Midwest (*n* = 1). Publication years ranged from 2015 to 2022, however more than half (*n* = 12) were published between 2020 and 2022. Only four articles had an explicit positionality statement by the author(s) (43–46); however, two of these had some relative ambiguity whether the positionality reported was that of the author(s) or if it was simply relaying the positionality of the instructor they were reporting on. Though only a small proportion of the articles had a positionality statement present, several others had authors note their perspectives, experiences, and expertise related to the research focus, thus demonstrating some reflexivity engagement (47).

### 3.1. Critical Race Theory

The most widely reported theory used within the identified articles was CRT. Articles categorized under this theory either utilized CRT as a whole or identified a specific tenet within CRT as its theoretical underpinning (i.e., centering the margins, power differentials, etc.). CRT frames race and racism as central, and specifically focuses on eliminating racism rather than ending race as an organizing principle (48), therefore articles that reported an explicit effort to address racism were also captured in this category. For example, Abuelezam and colleagues (49) sought to increase students’ ability to name race and racism as a social and structural determinant of health. One article conducted a systematic review to identify public health programs, curricula, and pedagogical methods that reify structural racism as a contributing factor to health disparities, social inequities, and structural issues (50). Complimentary to this review, the *American Journal of Public Health* published an editorial piece acknowledging Schools of Public Health’s efforts across the nation to engage student reflection in the structural inequalities and inequities African American populations face (51). They recognized a variety of work being done ranging from Tulane University, School of Public Health and Tropical Medicine’s organized lectures and panels, film screening, and performance to discuss concerns related to mass incarceration to Boston University, School of Public Health’s symposium on racism in housing and education, 400 Years of Inequality Timeline Activist Lab, and storytelling sessions.

As noted, some articles were more specific in the CRT tenets used to ground their work. One case is Fleming’s (52) effort to explain how a historical perspective on inequities is essential for public health researchers and practitioners to successfully reduce and eliminate health inequities. The author highlights their implementation of a three-credit, seminar-style elective to provide students a better understanding of historical policies, events, and movements that have led to health inequities, noting that course content, class activities, and classroom culture were developed through a lens of centering the margins, intersectionality, and awareness of power differentials. Similarly, Dimaano and Spigner (44) draw upon centering the margins and power differentials by utilizing a book-based seminar intervention where students read *The Immortal Life of Henrietta Lacks* by Rebecca Skloot to develop a more complex understanding of health disparities correlational relationship to the social determinants of health (i.e., socioeconomic status, religious beliefs, societal and institutional discrimination, access to health insurance, etc.). In doing so, students were more likely to cite race and racism as mechanism for why current health disparities exist.

### 3.2. Public Health Critical Race Praxis

This collection of articles presents a unique advancement in employing race theorization in public health, as they all utilize a well-established and widely cited framework that systematically and methodically combines CRT and public health. The PHCR praxis is an extension of CRT, as it infuses CRT tenets with public health practices through four identified focuses and ten guiding principles that imitate and expand upon the original CRT tenets (23, 24). This was the second most prevalent theoretical framework cited among the identified articles; however among these articles, the breadth of the PHCR praxis’ implementation and utilization is vast, demonstrating an opportunity to tailor its use for unique educational needs.

One article developed an entire academic institute around the PHCR praxis (53). The “Public Health Critical Race Praxis Institute” was a short-term, interactive training that engaged public health researchers, scholars, and postdoctoral fellows in reflective-based activities such as readings, presentations, dialogic engagement between peers and more. Ultimately, this training was considered a success due to several participants reporting that CRT, the crux of the PHCR praxis, is now a central tenet in their work, race is an important factor in health outcomes for their research, and they are actively seeking ways to employ CRT and PHCR praxis into their work. Upon the conclusion of the training, Butler III and colleagues (53) present several recommendations for others who wish to provide a similar organized training. These include (1) creating a sage space for individuals to be candid regarding their lived experiences, (2) ensuring a variety of instructional and pedagogical approaches, (3) maintaining connectivity with and among scholars, (4) providing networking activities among participants by region, health issue, or stage in schools and/or career, and (5) maintaining flexibility to adapt to emerging needs during the institute. McSorley and colleagues (46) work further validated these recommendations, though not as a result of an organized training, but as an organic, positive consequence of engaging with peers, proposing counter-curriculums, and fostering safe counter-spaces. The authors, who present a unique perspective due to their dual positions as doctoral students and college instructors, sought to link the content being discussed in their core public health courses to legacies of racism, colonialism, and other structural determinants of health as a means to evolve with student needs, societal momentum, and program commitments. In doing so, they created a working group of more than 10 graduate students that developed a counter-curriculum that included content on the theoretical and methodological topics currently omitted within public health curriculum such as U.S. colonial history, historical trauma, theories of embodiment, and alternative methodologies. Their efforts demonstrated a change in training environment that can serve as a model example of an actionable step to creating safe environments for authenticity, as well as opportunities to dismantle long-standing oppressive systems.

More specific pedagogical, instructional, and curricular models were presented by the remaining articles that employed the PHCR praxis. Among these includes Robillard and colleagues (54) work, represented by their thorough justification and recommendations for expanding the African American studies paradigm, to include public health. These authors cite relevant and successful programs and examples for their recommendations to infuse the PHCR praxis into course content, independent/directed studies, research and teaching assistantships, serving learning opportunities (i.e., internships, community partnerships), and seminars and conferences, allowing readers to build from their examples and implement successful pedagogical, instructional, or curricular models. The other article that offered a curricular model using the PHCR praxis was Lightfoot and colleagues (45) description of a creative, photography and written reflections-based activity employed in a Master of Public Health course to facilitate student exploration of racial identity. This article outlines a specific assignment students were tasked with, which asks students to create two photographic portraits to examine who they are or how they envision themselves, as well as a reflection on how they may be perceived within a racialized society. The assignment was found to foster a challenging, yet productive exchange of connection between students. Black, Indigenous, and People of Color (BIPOC) students were identified as readily being able to discuss their racialized identities compared to their White counterparts that found assignment to be a new and somewhat difficult experience.

Collectively, the described articles demonstrate the multi-level application of the PHCR praxis as a theoretical underpinning for organized trainings, organizational transformations/social movements, programmatic adaptations, and individual-based assignments.

### 3.3. Intersectionality

Intersectionality within itself is a theoretical and analytical framework for understanding and conceptualizing how multiple social identities, including race, gender, socioeconomic status, sexual orientation, disability, and more, intersect at the individual level of experience to reflect the interwoven systems of oppression and privilege at interpersonal, as well as societal and structural levels (55–58). Two articles explicitly note their use of intersectionality and social identity as a means to evaluate systems of power and privilege. One of these articles introduced the “Intersectionality Toolbox,” which grounded a public health course in intersectionality-based questions, concepts, and readings (59). In doing so, students reported a significant increase in generating knowledge, understanding diverse perspectives, and critically evaluating points of view, ultimately allowing these students to apply these concepts in a beneficial manner to support and serve the general public. This “toolbox” model provided a consistent instrument to refer back to throughout the semester, while also encouraging a recursive and iterative approach to perspective-taking among students. Comparatively, Njoku and Wakeel’s (60) work demonstrates instructors’ ability to translate faculty training, grounded in intersectionality, into public health curriculum. These workshops inspired faculty to infuse similar constructs into various public health courses, including both undergraduate and graduate coursework. The authors specifically note one assignment that was particularly successful engaging students in perspective taking and social identity. This activity, the “visualization activity,” asked students to illustrate how they envision health disparities through their own lens. This allowed students to exemplify creative and thought-provoking illustrations with the class, ultimately helping students emotionally connect with health disparities concepts, as well as create a sense of community with the shared reflections.

### 3.4. Antiracism

Articles that explicitly noted using an antiracist praxis as the theoretical and pragmatic underpinning of their efforts were unique in that none reported an individual assessment, but instead described a more holistic approach to competency reform (61), course design (62), and recognition of best practices (63). Antiracism has been introduced and coined as a racial concept that encompasses exterminating discrimination but also seeks to enable and promote the equality of racial and ethnic groups (64). Bentley and colleagues (63) were one of the two editorials identified, which is distinct from other articles in that it is not an active implementation of best practices, but instead an intentional presentation of others’ work that meets a journal’s call for papers. This article specifically presents a collection of articles identified by *Pedagogy in Health Promotion* that describe pedagogical practices used to incorporate concepts and topics of race, racism, social justice, and oppression within higher education classrooms. Works highlighted in this article presented a wide breadth of curricular, pedagogical, and instructional practices that engage students with antiracist principles such as the introduction and discussion of implicit bias with undergraduate public health students and the implementation of arts-based approaches to engage students in racial identity. Additional topics described include scientific racism, racial hierarchies and health outcomes, “hidden curriculum,” faculty perspectives on teaching anti-oppression concepts in the classroom, and minority student empowerment practices. Interestingly, some of the articles highlighted within this editorial were also included in this scoping review under different categories (45, 46). Though editorials are unique in that they often depict what a journal editor or editorial board is currently prioritizing, it demonstrates a disciplinary movement to attend to the journal’s call for antiracist work, while simultaneously presenting best practices for readers to readily implement.

Hagopian et al.’s (61) efforts are vast in that they present the manner in which their institution’s School of Public Health adopted and implemented an antiracist framework into their public health curricular competencies and college-wide cultural practices. Catalyzed by students’ demand for a more robust and “courageous” approach to race, the college committed to restructuring its competencies to better align with antiracist frameworks. In doing so, active members of this movement underwent training to address issues of equity and diversity at University of Washington School of Public Health (UWSPH), developed a work plan, adjusted competency language, and implemented the new antiracist competencies. Continued efforts are reported to maintain this competency framework through program evaluations and corresponding recommendations to further their implementation of antiracism within the college such as adding questions regarding classroom climate to course evaluation forms and adding language regarding classroom climate to course syllabi. The final article that centers antiracism is a presentation of an antiracist pedagogical approach as the crux of a graduate assessment and planning course design and implementation (62). The authors describe course components, which primarily explain instructional strategies and course assessments. They specifically note that instructors utilized nonfiction literature, change experts/practitioners, case-based teaching, and community-based projects to engage students in an antiracist pedagogy. Reflections and team-based projects were used to assess students’ engagement with and understanding of racial topics such as interpersonal, structural, and systematic racism. Their description is particularly unique in that it accentuates the compatibility and cohesiveness these instructional strategies and assessments have when applied tangentially, noting that any separation may diminish the success of their reported outcomes.

### 3.5. Contemplative pedagogy and critical service-learning/community engagement

The following articles explicitly note utilizing a pedagogical approach to cultivate learning environments that foster open awareness, introspection, authentic connection, and community engagement in the hopes of providing students with insight into systems of privilege and oppression (43, 65, 66). Pedagogy differs from instruction and curriculum due to its central focus being on teaching practices and the organized mechanisms that are intentionally employed to engage students in specific content matter (67). Therefore, in this categorization of results, a specific attention toward the organization and recommendation of course structure in addition to the curriculum being delivered was present. Batada (43) describes the context and structure of a 200-level course, *Health Parity: Domestic and Global Contexts*, at a state public liberal arts university, while also presenting various mechanisms to introduce contemplative practices to students. In doing so, they note that the course is divided into three main sections: (1) Identities and Health: Construction and Measurement; (2) Health Disparities Across Class, Race/Ethnicity, Sexuality, Gender, and Intersecting Lines of Difference; and (3) Trends in Global Health. All three sections emphasize learning outcomes related to the social construction of identities, expression of experiences, power dynamics, intersectionality, and critical consciousness. To reach these learning outcomes, the author identifies relevant contemplative pedagogy concepts such as attention, interpretation, nonjudgment, holding multiple truths/sustaining contradictions, interconnectedness, reflection, compassion, and solidary, all of which are integrated into various learning activities and course design elements.

Though Batada (43) described course resides in a traditional classroom setting, they recognize that social justice education, such as public health, involves community engagement, which is emphasized by the other two identified articles through their particular focus on critical service-learning (65, 66). Levin and colleagues (66) provide a holistic review of best practices and emerging innovations in community engagement across public health education, research, and practice. In doing so, they emphasize the value of critical serving-learning within education-based practices. They note that this pedagogical approach simultaneously develops public health students’ knowledge, skills, and competencies through “field placements,” ultimately allowing students to focus on social transformation through intentional efforts to examine and challenge power, privilege, and systems of oppression. Derreth and Wear’s (65) efforts echo several of these sentiments but add a unique integration of critical service-learning through online mediums. They describe an online public health doctoral course, which sought to connect the three main parts of service-learning: academic knowledge, reflective discussion, and community collaboration. A significant portion of the course was composed of the community project students and community-based organizations (CBOs) collaborated on, thus allowing practice application of theoretical concepts. Upon the conclusion of the course, the instructors found that the work students and CBOs collaborated on provided the CBOs with long-term, usable tools, students took great care in understanding the community needs, and making a space for reflection, alongside prioritizing active collaboration brought a shared meaning and urgency to the course.

### 3.6. Ethnic studies

Only one article reported using an ethnic studies framework, which seeks to “rehumanize individuals through the acknowledgment and validation of their experiences as sites of epistemological inquiry, challenge and decenter Eurocentric narratives and perspectives, and foster ongoing intersolidarity movements of BIPOC communities and White accomplices for racial justice and self-determination” (68). The authors describe their use of an ethnic studies framework while teaching an Asian American Community Health Issues course, while simultaneously justifying the opportunity ethnic studies provide for instructors and students to recognize, affirm, and collectively act on the needs and concerns of various communities when included in public health and health education coursework. The case example presented by Maglalang and colleagues (68) demonstrates a mechanism and theoretical grounding for instructors to position racialized issues (i.e., discriminatory violence, health inequities, etc.) within larger historical, social, and political contexts, while also demonstrating to students the self-determination of communities of color and their responses to organize and prioritize their community’s health. This framework, therefore, provides a lens of empowerment rather than victimization, allowing students to analyze and unpack the current conditions of a variety of racial and ethnic groups through a strength-based model.

## 4. Discussion

In 2010, CEPH called for accredited schools and programs of public health to demonstrate a commitment to diversity (29, 30), while Ford and Airhihenbuwa (23, 24) simultaneously presented the first working praxis that integrated CRT into the public health domain. In doing so, these germinal efforts should serve as catalysts for social transformation in public health, particularly within public health education due to CEPH’s significance and influence within academia. To assess where educational efforts have gone since this initial call, this scoping review asked: Since 2011, how have faculty and instructors published their use of race theorization in public health curriculum/instruction within the United States?

Though there is a significant push toward addressing and dismantling racism through public health efforts, the present scoping review found only 18 examples in peer-reviewed literature within the last decade that explicitly provide exemplary models and/or scholarly recommendations grounded in critical race theorizations within public health pedagogy, instruction, or curriculum. However, among the identified articles, a wide breadth of innovative approaches to infusing critical race studies within public health higher education was shown. These approaches range from individual assignments to course design and implementation to institutional culture change, thus demonstrating the multifaceted nature of critical race studies within micro-learning communities and macro-discipline practices. Among the articles that centered around individual assignments (44, 45, 49, 60), a collective attention toward engaging students’ critical consciousness was noted (69). Though the origin of critical consciousness frames how marginalizes communities and individuals analyze oppressive and discriminatory systems (69, 70), a cornerstone of this theoretical construct is critical motivation and action to dismantle systems of oppression and seek social justice. As such, the employment of critical consciousness can and should encompass both marginalized communities, as well as their co-conspirators. As such, one leg of critical consciousness is critical reflection, which was ubiquitous across the described assignments and activities. By engaging students in this critical reflection, it fosters students to be racially conscious (23), but also recognizes other social identities that may influence their position within American society (55). As such, Robillard and colleagues (54) even go as far to say that fostering such critical consciousness may be a better approach toward teaching health equity than traditional practices.

Another notable contribution to the success of these curricular, pedagogical, and instructional efforts was the emphasis on co-constructing group norms and standards, which borrows from constructivist and social constructivist principles (71). Due to this scoping review focusing on institutions of higher education, several authors approached cultivating inclusive and safe learning environments by collaborating with their students at the start of the course (52, 59), while others recognized the importance of fostering these environments to foster candid sharing and reflection among peers (46, 53). Co-constructing group norms and standards to allow for a safe space to develop authentic relationships and engage in critical reflection borrows from various research principles such as participatory action research or community based participatory research, which seek to emphasize a collaborative effort toward social transformation, advocate for shared power, and involve participants in the research process (72). Though students are not engaging in a “research” process, they are still key stakeholders vulnerable to the power dynamics that may inadvertently enter a classroom space. Furthermore, practices of co-constructing assessment instruments have been found to increase student motivation and enhance their confidence in completing tasks (73). Therefore, by collectively establishing group norms, it combats power dynamics and feelings of isolation by reifying the vision of the classroom community and making it explicit, while also likely increasing student motivation to engage in conversations about race and racism.

One noticeable gap in the identified articles is when specific racial groups were discussed concerning discrimination and racism, it often focused on Black and African American populations and their experiences. Though there were some differentiating perspectives, such as Maglalang and colleges’ (68) article based on teaching an Asian American Community Health course, McSorley and colleagues’ (46) disclosure of being descendants of either Puerto Rican or Filipino ancestry, and Batada’s (43) positionality statement of her South Asian descent, there was a complete absence of Indigenous population perspectives. This was largely due to articles identified as relevant in terms of public health education being deemed ineligible during screening stages due to taking place outside of the United States. For example, one article addressed knowledge gaps in Indigenous public health by critiquing current Master of Public Health competency standards from the perspective of an Indigenous public health graduate (74), but this work was conducted in Australia. Other accounts of Indigenous population health curriculum are recognized in the *Australian Journal of Indigenous Education* upon their call for literature that situates the role of higher education in building an Indigenous health workforce (75), but again, this work resides outside of the United States. Other articles that centered their focus on Indigenous public health were noted in New Zealand and Canada, consequently exacerbating the dearth of Indigenous health included in American curriculum. As such, public health education literature may inadvertently perpetuate “hierarchies of oppression” by recognizing one group’s oppression more significantly than another’s, thus decreasing the hurt, violation, and victimhood Indigenous populations have endured (48). It is only when ethnic and racial groups have been recognized and attended to in equitable solidarity will we finally start making strides toward true social justice for all.

It is essential to recognize that more than half of the identified articles were published after 2020 (45, 46, 50, 51, 59, 62, 63, 65, 66, 68). Chandler (50) specifically acknowledges that “the last decade has seen calls for more systematic and rigorous approaches to public health pedagogy” (50) citing various calls for competency overhauls (76, 77) and cultivating learning communities (78); however, we would be remised to not call into question that it took significant social movement catalyzed by great loss to finally initiate appropriate research and scholarly engagement with these topics. Due to the late onset of these publications, the snowballing methodology used in this scoping review produced only one additional article likely due to four of the ten articles identified as “ideal candidates” have not received any reported citations (43, 45, 46, 52). The lack of citation is likely due to their recency. Though the influence of social context cannot be understated, it should not be the only catalyst when a wide variety of key stakeholders, including students, professional organizations, and accrediting bodies, are calling for scholars to engage in this work.

### 4.1. Limitations

There are several limitations to consider when discussing this scoping review. Though this scoping review utilizes an expansive list of key search terms to capture critical race theorizations, it is recognized that several widely used theories were not included. The search terms selected were meant to ensure the widest breadth of critical race theorizations were captured rather than being the most exhaustive list of key search terms. Another limitation of this study is that it strictly sought to explore instructor and/or faculty perspectives on public health education practices. This omits the unique and meaningful perspectives of other key stakeholders such as public health students and community members. Furthermore, this scoping review excluded articles that strictly presented a call to action, rather than an exemplary model for practice. Though the authors recognize calls to action as powerful cues to catalyze change, they may never translate from cognitive ambitions to tangible actions and models. Therefore, in excluding articles that strictly present calls to action, this scoping review provides readers access to exemplary models and actionable practices to readily implement and expand upon in their own work. Lastly, although this scoping review used rigorous and systematic procedures to comprehensively review available peer-reviewed literature, articles that may have addressed the research question may have been missed due to being identified as legal articles, dissertations, gray papers, non-peer-reviewed journal articles, books, or reports. Similarly, by not collecting curricular documents that speak to programmatic structures, curricular frameworks, overarching themes, and instructional content, another facet of this work may be inadvertently missed. In recognition of this, the authors note that public health and health education faculty may in fact be engaging in this work, but simply not publishing their efforts. This could be due to a variety of factors such as tenure and promotion requirements, perceived rigor of pedagogical research compared to other forms of scholarship, and more. Therefore, we recommend that future reviews search additional databases and sources, particularly conference presentations and reports, because many active instructors may reserve sharing their pedagogical, instructional, and curricular practices for more informal and interactive settings such as conferences or working groups. In addition, we encourage academic institutions to review their criteria (i.e., tenure and promotion standards) to support the need for pedagogical and scholarly activity and publication to continue to advance the field forward.

## 5. Conclusion

This scoping review contributes to the limited working body of public health education literature that utilizes critical race theorizations. Identifying theoretically grounded, exemplary models and scholarly recommendations of pedagogical, instructional, and curricular practices provides readers to borrow from successful practices and implement concepts of race, racism, antiracism, intersectionality, and more into their classrooms. The articles identified as descriptive best practices can be especially helpful in assisting instructors and faculty in developing holistic courses, implementing various assignments and activities, and fostering inclusive learning communities. Public health scholars should learn from these articles that work on infusing critical race studies into their educational efforts; however, they should also continue to strive to implement new innovative ways to permeate critical race theories throughout their curricular, pedagogical, and instructional strategies. By doing so, public health education will not only meet CEPH’s call to demonstrate a commitment to diversity (29, 30), but also make active strides alongside other disciplines in dismantling current discriminatory and oppressive systems to achieve social justice and health equity.

## Author contributions

SC contributed to preliminary discussions regarding research design and corresponding methodologies, conducted the data collection and review procedures, charted and summarized the data results, and wrote the introduction, methods, results, and discussion portions of this manuscript. TS contributed to preliminary discussions regarding research design and corresponding methodologies and conducted review procedures as the second reviewer alongside SC. GH and MM contributed to preliminary discussions regarding research design and corresponding methodologies. All authors reviewed and edited the manuscript in its final stages.

## Funding

The use of Covidence was supported by the University of Florida Clinical and Translational Science Institute, which is supported in part by the NIH National Center for Advancing Translational Sciences under award number UL1TR001427. The content is solely the responsibility of the authors and does not necessarily represent the official views of the National Institutes of Health.

## Conflict of interest

The authors declare that the research was conducted in the absence of any commercial or financial relationships that could be construed as a potential conflict of interest.

## Publisher’s note

All claims expressed in this article are solely those of the authors and do not necessarily represent those of their affiliated organizations, or those of the publisher, the editors and the reviewers. Any product that may be evaluated in this article, or claim that may be made by its manufacturer, is not guaranteed or endorsed by the publisher.

## Supplementary material

The Supplementary material for this article can be found online at: https://www.frontiersin.org/articles/10.3389/fpubh.2023.1148959/full#supplementary-material
