# Supplementary material for: Exploring public health education’s integration of critical race theories: A scoping review
Source: Front Public Health. 2023 Apr 14;11:1148959. doi: 10.3389/fpubh.2023.1148959 (PMC10140291; doi:10.3389/fpubh.2023.1148959)
Supplement: Supplementary file 1 [file Table_1.DOCX]

Table 1. Summary of Included Articles

| **Manuscript Title / Manuscript Type** | **Author(s), Year / Region/State of Study** | **Reported Critical Race Theorization** | **Aims/Purpose** | **Key Characteristics & Findings** |
| --- | --- | --- | --- | --- |
| Naming racism in the public health classroom / Original Research | Abuelezam et al., 2020 / Northeast Region | Racism - ability to name race and racism | Explore the impact of local and national events on students' ability enrolled in an introductory public health course at a predominately White institution to name racism and highlight the impact of racism on maternal and infant health disparities. | Instructors utilized a PBS documentary to exemplify health disparities between Black and White mothers in the United States, followed by an instructor-led discussion emphasizing racism as a primary determinant for this disparity. These course materials and instructional strategies were the cornerstone to building students' skills in naming racism. However, results show that national events did not seem to impact students' ability to name racism or provide alternative explanations, whereas local campus-level events did have a positive correlation with student responses naming racism and providing alternative explanations. Embedding local climate into public health teaching may impact students' learning about social and structural determinants of health. |
| Utilizing Contemplative Practices With Undergraduate Students in a Community-Engaged Course on Health Disparities / Descriptive Best Practices | Batada, 2018 / North Carolina | Contemplative Pedagogy - cultivating mechanism for open awareness and introspection regarding privileged and oppressed identities | Describe (1) the context and structure of a 200-level course, Health Parity: Domestic and Global Contexts, at a state public liberal arts university, (2) mechanisms to introduce contemplative practices to students, and (3) exemplary student learning activities. | The course is divided into three main sections: (1) Identities and Health: Construction and Measurement; (2) Health Disparities Across Class, Race/Ethnicity, Sexuality, Gender, and Intersecting Lines of Difference; and (3) Trends in Global Health, with all emphasize learning outcomes related to the social construction of identities, expression of experiences, power dynamics, intersectionality, and critical consciousness. To reach these learning outcomes, the author identifies relevant contemplative pedagogy concepts such as attention, interpretation, non judgment, holding multiple truths/sustaining contradictions, interconnectedness, reflection, compassion and solidary which are embedded through various learning activities to train students to ask questions of themselves in their work and work with others. |
| Antiracism and the Pursuit of Justice / Editorial | Bentley et al., 2021 / N/A | Antiracism | Present a collection of articles that address pedagogical concepts and practices that explicitly focus on antiracism and its impact on health and social justice. | A collection of articles were identified by *Pedagogy in Health Promotion* that describe pedagogical practices which incorporate concepts and topics of race, racism, social justice, and oppression within higher education classrooms. One article explained innovative ways to introduce and discuss implicit bias with undergraduate public health students. Other articles touch on mechanisms, such as arts-based approaches, to engage students in racial identity. Additional topics presented within the selected articles include scientific racism, racial hierarchies and health outcomes, "hidden curriculum," faculty perspectives on teaching anti-oppression concepts in the classroom, ways to best support community partners through collaborative, communicative, and reflective practices, minority student empowerment practices, and curricular responsive review model to incorporate health equity into public health curriculum. |
| Critical Race Theory Training to Eliminate Racial and Ethnic Health Disparities: The Public Health Critical Race Praxis Institute / Commentary | Butler III et al., 2018 / Maryland | Public Health Critical Race Praxis - Critical Race Theory | Describe an innovative academic institution training experience, the Public Health Critical Race Praxis Institute, which focuses on instructing researchers and researcher-in-training on pragmatic methods for adoption of a Public Health Critical Race Praxis framework and utilization of Critical Race Theory in their research. | The Institute was held over two and a half days, which utilized interactive and dynamic approaches (i.e., readings, activities, presentations, reflections and dialogue between peers) to focus on using the Public Health Critical Race Praxis (PHCRP) and Critical Race Theory (CRT). The Institute was considered a success due to several participants reporting that CRT is now a central tenet of their work; race is an important factor in health outcomes in their research; and they are seeking ways to employ CRT and the PHCRP into future projects. Some lessons learned include extending the duration of the Institute, conducting booster sessions at regular intervals to reinforce the information, and implementing other sources of information such as cultural studies and history to triangulate and strengthen information delivery. |
| Training Public Health Students in Racial Justice and Health Equity: A Systematic Review / Systematic Review | Chandler et al., 2021 / N/A | Racism - Reifying structural racism | Synthesize peer-reviewed literature through means of systematic review, that describe programs, curricula, and pedagogical methods designed to train students attending schools and programs of public health within the United States in structural racism and the application of racial equity principles to public health practice and scholarship. | 11 articles were identified that met study eligibility criteria. These 11 articles framed several key instructional topics such as health disparities or inequities, structural issues, social determinants of health, racism or antiracism, social justice, health, and pedagogy of collegiality (i.e., application of critical theories in classroom activities). Instructional programs ranged from in-person classes to workshops or seminars. The limited peer-reviewed literature identified in this review implicates public health education because schools and programs of public health have little pedagogical guidance available to them when hoping to adapt their programs to center race and racism. Furthermore, there is little consensus on how to teach about racism most effectively. This continues to leave scholars ill-equipped in course and curricular design. However, pedagogical, and instructional efforts surrounding race and racism are still in their infancy. |
| Critical Online Service-Learning Pedagogy: Justice in Science Education / Descriptive Best Practices | Derreth & Wear, 2021 / Maryland | Critical Service-Learning as a means of connecting with the local community to explore and address issues of diversity, racism, and social justice | Explore a critical online service-learning framework, grounded in social change, authentic relationships, and redistribution of power, and offer an example of a philosophy-based public health science doctoral course that used this framework. | The online public health doctoral course was designed to encourage dialogic communication through (1) lectures, (2) podcast-style recordings of conversations to provide practice-based examples and reflection, and (3) "project checkpoints" or activities for practice of theory application. These communication modes sought to connect the three main parts of service learning: academic knowledge, reflective discussion, and community collaboration. The bulk of the course was composed of the community project students and community-based organizations (CBOs) collaborated on, allowing students to actively engage and practice course-based knowledge and assignments. Upon the conclusion of the course, the instructors found that the work students and CBOs collaborated on provided the CBOs with long-tern, usable tools; students took great care in understanding the community needs; and making a space for reflection, alongside prioritizing active collaboration brought a shared meaning and urgency to the course. |
| Teaching from the Immortal Life of Henrietta Lacks: Student perspectives on health disparities and medical ethics / Original Research | Dimaano & Spigner, 2017 / Washington | Racism - Social Determinants of Health; Health Disparities; Structural Racism; Medical Ethics | Assess Master of Public Health students' perceptions of health disparities and medical ethics before and after a book-based seminar intervention using The Immortal Life of Henrietta Lacks by Rebecca Skloot. | 14 Master of Public Health students were assigned to a book-based seminar course (intervention group) and 3 students acted as the non-intervention group over a 10-week period. Qualitative results, that utilized thematic analysis, found that all students were aware of health disparities over the course of the 10 weeks, however, the intervention group gained a more complex understanding of health disparities correlational relationship to the social determinants of health (i.e., socioeconomic status, religious beliefs, societal and institutional discrimination, access to health insurance, etc.). More specifically, the intervention group was more likely to cite race and racism as mechanisms for why current health disparities exist. |
| The Importance of Teaching History of Inequities in Public Health Programs / Perspective | Fleming, 2020 / Michigan | Critical Race Theory - Centering the Margins; Intersectionality; Power Differentials | Explain how a historical perspective on inequities is essential for public health researchers and practitioners to successfully reduce health inequities and present an example course titled, "Historical Roots of Health Inequities," to showcase a model to potentially include in other public health programs. | A three-credit, seminar-style elective was created to provide students a better understanding of historical policies, events, and movements that have led to health inequities. The course engages students in readings from a variety of professions (i.e., history, law, and more rather than just public health authors), short reflections, and classroom discussions. For discussions to be successful, the instructor accentuated the importance of creating an inclusive and participatory classroom environment, which was achieved by collectively creating group norms. To connect historical inequities to their present-day impact, without leaving students feeling hopeless, the instructor utilized two instructional activities during each class session: (1) a public health skills workshop for undoing structural inequities, and (2) student-led presentations on resistance movements. Though only a 12-week course, students had the opportunity to explore various avenues they can pursue within the public health field to address historical inequities and explore various mechanisms already being utilized to undo structural inequities. |
| Adopting an Anti-Racism Public Health Curriculum Competency: The University of Washington Experience / Descriptive Best Practices | Hagopian et al., 2018 / Washington | Antiracism | Present and reflect upon the manner in which the University of Washington adopted and implemented an antiracist framework into their public health curricular competencies and college-wide cultural practices. | The University of Washington, School of Public Health (UWSPH) committed to restructuring its competencies to better align with antiracist frameworks. In doing so, active members of this movement underwent a training to address issues of equity and diversity at UWSPH, developed a workplan, adjusted competency language, and implemented the new antiracist competency. The UWSPH continues to maintain this competency framework through evaluation efforts and making updated recommendations to further their commitment to antiracism. The authors note that larger social forces influenced stakeholders' willingness to commit to these institutional changes, as well as local, racially-charged incidents and powerful student voices. In addition, they state that developing a specific subcommittee solely dedicated to upholding these anti-racist practices was essential. |
| Enhancing Community Engagement by Schools and Programs of Public Health in the United States / Literature Review | Levin et al., 2021 / N/A | Critical Service-Learning - Community Engagement, Centering the Margins, Community Voices; Racism - Structural Racism and Racial Inequity | Review best practices and emerging innovations in community engagement for public health education, research, and practice such as critical service-learning, community-based participatory research, and collective impact to ultimately overcome challenges face when working with historically marginalized communities such as mistrust by community members, imbalance of power, and unequal sharing of credit. | The authors conducted a literature review to identify the best practices to engage community organizations and members. They identified three overarching pillars: (1) education, (2) research, and (3) practice. Education-based practices included critical-service-learning, practicum requirements, and social justice and racism content. Research-based findings included community-based participatory research methodologies. And finally, practice included implementing collective impact approaches. In addition to these three pillars, the authors present more global best practices such as institutional efforts and culture change efforts. The authors argue that to overcome the mistrust that community members current have toward public health entities due to the longstanding inequities and unjust actions such as the Tuskegee Syphilis Study, public health researchers, practitioners and policymakers must demonstrate trustworthiness in their actions. The practices identified in this article are the first steps in doing so. |
| Developing an Antiracist Lens: Using Photography to Facilitate Public Health Critical Race Praxis in a Foundational MPH Course / Descriptive Best Practices | Lightfoot et al., 2021 / North Carolina | Public Health Critical Race Praxis - Critical Race Theory | Offer a curricular model that integrates the Public Health Critical Race Praxis with a creative, photography and written reflections-based approach, to facilitate exploration of racial identity among first year Master of Public Health students. | As part of an effort to facilitate exploration among Master of Public Health students of how factors such as race as a social construct and racism as a critical determinant of health outcomes affect their role as a future public health professional, the instructors offer a racial identity assignment. This assignment asks students to create two photographic portraits: (1) an examination of who they are and or how they envision themselves, and (2) a reflection on how they may involuntarily be perceived in a racialized world. Through this assignment, both White and BIPOC students see the unique ways that their racial identities influence their ability to conduct meaningful public health work. Among the participating BIPOC students, they are readily able to discuss their racialized identities as important aspects of their lived experiences and convey a fluidity in regard to their racialized identities. In contrast, the experience of grappling with their racial identity was new for a majority of the participating White students. This activity has been identified as a creative approach and tool toward shifting the classroom racial dynamic that fosters a challenging, yet productive exchange of connection, rather than discord or tension among various racial identities. |
| Centering Ethnic Studies in Health Education: Lessons From Teaching an Asian American Community Health Course / Perspective | Maglalang et al., 2021 / California | Ethnic Studies Framework | (1) Demonstrate that employment of ethnic studies in public health curricula presents an opportunity for instructors and students to recognize, affirm, and collectively act on the needs and concerns of various communities and (2) describe lessons learned from teaching an Asian American Community Health Issues course that integrates an ethnic studies lens to understand Asian American community health. | Utilizing an ethnic studies lens within public health and health education coursework allows instructors to position racialized issues (i.e., discriminatory violence, health inequities, etc.) within larger historical, social and political contexts. Furthermore, grounding coursework in this framework allows instructors to position materials so that students can recognize the self-determination of communities of color and their responses to organize and prioritize their community's health, thus providing a lens of empowerment rather than victimization. Implementing an ethnic studies lens was found to raise the critical consciousness of students, allowing them to analyze and unpack current conditions of a variety of racial and ethnic groups through a strength-based model. |
| Doctoral Students as Agents for Change: Shaping Our Public Health Training Environment / Perspective | McSorley et al., 2021 / California | Public Health Critical Race Praxis - Critical Race Theory; Critical Pedagogy | Share the pedagogical mechanisms in which three doctoral student instructors applied critical strategies to identify gaps in public health training, reflect on the value of lived experiences and ancestral histories, and take action toward advancing change in public health education. | A group of doctoral students, who double as course instructors, sought to link the content being discussed in their core public health courses to legacies of racism, colonialism, and other structural determinants of health. In doing so, they created a working group of more than 10 graduate students that developed a counter-curriculum that included content on the theoretical and methodological topics currently omitted within public health curriculum such as U.S. colonial history, historical trauma, theories of embodiment, and alternative methodologies. This group then conducted a qualitative evaluation protocol to investigate the barriers and facilitators to implementing health justice curriculum. To further their efforts, they connected with various key stakeholders including the School of Public Health's Faculty Curriculum committee and the school-wide Equity, Diversity, and Inclusion committee. These efforts demonstrated a change in training environment and serve as an example of an actionable approach to create counter spaces and dismantle long-standing oppressive systems. |
| Infusing Health Disparities Awareness Into Public Health Curricula at a Rural Midwestern University / Descriptive Best Practices | Njoku & Wakeel, 2019 / Midwest | Health Disparities through the lens of social identity, intersectionality, power, and privilege | Describe faculty efforts, including course design, delivery, and evaluation, for health disparities-related education development in both undergraduate and graduate public health courses | Five faculty members were tasked to develop new courses for two public health programs (one undergraduate and one graduate). These faculty members underwent various teaching development programs, which inspired and motivated faculty to employ instructional and pedagogical approaches to enhance student learning about health disparities. Course learning outcomes, lecture topics, and course objectives were designed to build global awareness of health disparities, alongside specific learning activities to supplement the engagement with the health disparities content. Upon the conclusion of course implementation, course evaluations and teaching observations found that students expressed higher feelings of burn out and less engagement if health disparities were addressed in the latter part of the semester. However, one activity was found to be particularly engaging among students. This activity, the "visualization activity," provided students with crayons and sketch paper and asked them to illustrate how they envision health disparities through their own lens. Students who were traditionally less engaged were found to share more creative and thought-provoking illustrations with the class. In addition, this activity was thought to help students emotionally connect with health disparities concepts, as well as create a sense of community with the share reflections. Other student reflection-based assignments were found to also encourage strong introspection of health disparities-related content. |
| Expanding the African-American Studies Paradigm to Include Health: a Novel Approach to Promoting Health Equity / Brief Report | Robillard et al., 2015 / N/A | Public Health Critical Race Praxis - Critical Race Theory | Offer justification and recommendations for expanding the African-American studies paradigm using a public health-oriented approach to better attend to and examine health disparities and the social determinants of health. | The authors make an argument for the advantageous nature of combining public health, health education, and African-American studies. In doing so, they propose several recommendations to incorporate this intersection in disciplines within current curriculum and training, citing relevant and successful programs/examples for each recommendation. These include course content, independent/directed studies, research and teaching assistantships, serving learning opportunities (i.e., internships, community partnerships), and seminars and conferences. More specifically, the authors present two models to assist in the paradigm shift: (1) an interdisciplinary (or multidisciplinary) model and (2) a transdisciplinary model. |
| [Un]Forgetting History: Preparing Public Health Professionals to Address Structural Racism / Descriptive Best Practices | Rosario et al., 2022 / North Carolina | Antiracism - Antiracist Pedagogy | Present an antiracist pedagogical approach used within a Master of Public Health assessment and planning course and outline how this approach empowers students to name structural racism, understand how it operates within society, and build skills to take action. | Instructors utilized nonfiction literature, change experts/practitioners, case-based teaching, and community-based projects to engage students an anti-racist pedagogy. Reflections and a team-based project were used to assess students’ engagement with and understanding of racial topics such as interpersonal, structural, and systematic racism. Using nonfiction literature was found to fill knowledge gaps and reify racial experiences, depicting the ordinariness and depth of racism, as well as its extensive consequences. Case-based examples paired with change agents demonstrated a shift in how students named and described racism, noting after the implementation of these pedagogical and instructional efforts, that explicit mechanisms in which racism and racialized oppression perpetuate health disparities. Finally, the community-based project provided a collaborative opportunity to apply concepts and proposal sustainable solutions to undo structural racism. |
| The Intersectionality Toolbox: A Resource for Teaching and Applying an Intersectional Lens in Public Health / Descriptive Best Practices | Sabik, 2021 / Rhode Island | Intersectionality | Describe the 'Intersectionality Toolbox,' a framework developed from a variety of resources to apply an intersectional perspective to public health issues, and how to implement it in public health courses. In addition, outline the results of an evaluation of the 'Intersectionality Toolbox' in an undergraduate public health course. | Upon the conclusion of implementing the 'Intersectionality Toolbox' in a public health course, student evaluations showed that "students reported a significant increase in gaining factual knowledge about the topic, developing knowledge, understanding diverse perspective, applying course material, developing competencies and skills needed in the profession, learning to find and evaluate resources, critically evaluating points of view, and applying knowledge to benefit others and serve the public good" (p.5). Students favored the intersectional lens being incorporated, as many indicated they would change "nothing" about the course. Finally, students provided positive feedback to discussing examples and walking through them in class to apply 'Intersectionality Toolbox' questions to a variety of public health and healthcare settings. |
| Observing an Anniversary: The 400 Years of Inequity Project / Editorial | Sember et al., 2021 / N/A | Racism - Structural Inequality | Present the work public health instructors, researchers, and practitioners have been conducting as a result of the 'Inequity Project,' including how various Schools and Programs of Public Health have integrated conversations regarding structural inequalities in the United States. | In an editorial presentation of the 400th anniversary of the arrival of the first Africans in Jamestown, reviewers identified and present various Schools of Public Health's effort to engage student reflection in the structural inequalities and inequities African Americans face. Amongst the identified efforts include Tulan University, School of Public Health and Tropical Medicine's organized lectures and panels, film screening, and performance to discuss concerns related to mass incarceration; Mailman School of Public Health's musical performances , grand round presentations and symposia on medical apartheid, Boston University, School of Public Health's symposium on racism in housing and education, 400 Years of Inequality Timeline Activist Lab, and storytelling sessions; Harvard University, T.H. Chan School of Public Health's medical racism and chattel slavery discussions; and finally, Drexel University, Dornsife School of Public Health's preconference session for the Society for the Analysis of African American Public Health Issues to discuss public health's response to inequities faced by communities in African Diaspora. |
